# Multifractality and cross-correlation analysis of streamflow and sediment fluctuation at the apex of the Pearl River Delta

**DOI:** 10.1038/s41598-018-35032-z

**Published:** 2018-11-08

**Authors:** Yao Wu, Yong He, Menwu Wu, Chen Lu, Shiyou Gao, Yanwen Xu

**Affiliations:** 1 Key Laboratory of the Pearl River Estuarine Dynamics and Associated Process Regulation, Ministry of the Water Resources, Guangzhou, 510610 China; 2 Pearl River Hydraulic Research Institute, Pearl River Water Resources Commission, Guangzhou, 510610 China; 3 College of Harbor, Coastal and Offshore Engineering, Hohai University, Nanjing, 210098 China

## Abstract

The fluctuation and distribution of hydrological signals are highly related to the fluvial and geophysical regime at estuarine regions. Based on the long daily streamflow and sediment data of Makou (MK) and Sanshui (SS) stations at the apex of the Pearl River Delta, the scaling behavior of the streamflow and sediment is explored by multifractal detrended fluctuation analysis (MF-DFA). The results indicated that there was significant multifractal structure present in the fluctuations of streamflow and sediment. Meanwhile, the multifractal degree and complexity of sediment were much stronger than streamflow. Although the scaling exponents of streamflow were larger than sediment at both MK and SS, no evident differences have been found on the scaling properties of streamflow and sediment for the ratios MK/SS. Moreover, the cross-correlation between streamflow and sediment is further detected by Multifractal Detrended Cross-Correlation Analysis (MF-DXA). The multifractal response between streamflow and sediment at small timescale is characterized by long-range correlations whereas it exhibits random behavior at large timescale. The interaction of the broadness of probability density function and the long-range correlations should be responsible for the multifractal properties of hydrological time series as the multifractal degree of surrogate and shuffled data was significantly undermined.

## Introduction

The fluctuation of fluvial dynamic and sediment structure, associated with the identification of various geophysical and hydrological characteristics, can exert substantial control on the natural system of alluvial deltas and estuaries^1,2^. Due to distinct regional regime and anthropic interference, the hydrological phenomena generally display self-affine and self-similar fractal behaviors over multiple time scales^3^. As recommended by the National Research Council, substantial attention should be paid to the invariance property across scales in the hydrological processes, which contribute considerably to the management of water resource and the predication of morphological evolution^4,5^.

The streamflow fluctuation with dynamical input (precipitation) and output (evaporation) is generally characterized by long-range power-law correlation. Analogously, the sediment fluctuation is closely intertwined with the pattern of depletion (deposition) and supply (proximal/distal erosion)^6,7^, implicating the fractal properties of the underling sediment dynamics^8^. Exploration and determination of such correlation are of help to the understanding of the intrinsic behavior of the corresponding hydrological fluctuation and to predict their future events. More than half a century ago, Hurst first proposed that the annual streamflow records in the Nile River exhibited “long-range statistical dependencies”. Similar long-range correlations have also been found in a remarkably wide variety of natural phenomena in later researches^9,10^. However, the intrinsic fluctuations of hydrological process are fairly vulnerable to the non-linearity and non-stationarity such as artificial noises and trend patterns, which may lead to spurious or at least unreliable results in the analysis of the long-range correlations. Hurst’s original R/S analysis was thus criticized, since it failed to distinguish trend from the hydrological fluctuations^11,12^.

In order to obtain a robust detection of the long-range correlations associated with the fractal behavior of the hydrological alteration, detrended fluctuation analysis (DFA) was introduced to analyze non-stationary time series in the presence of trend^13^. Although such monofractal approach proved to be useful, a full characterization of the fluctuations was required on the fractal properties studies^14^, because as a modified version of DFA, the multifractal detrended fluctuation analysis (MF-DFA) allows a reliable multifractal characterization of the fluctuation pattern^15^. This multifractal description can be regarded as a “fingerprint”, which has been widely applied to a wide range of fields, including pathological states in biomedical signals^16,17^, stock market efficiency in financial records^18^, streamflow and precipitation in hydrological data^3^. Furthermore, since two synchronous time series, recorded temporally or spatially in the natural system, have the potential to be cross-correlated and possess multifractal characteristics, Multifractal Detrended Cross-Correlation Analysis (MF-DXA) was thus introduced by Zhou to explore the power-law cross-correlations between two non-stationary series with multiple orders detrended covariance^19^. Based on the state-of-the-art algorithm of MF-DXA, long-range cross-correlation between the underlying streamflow data and typical climate criteria, such as precipitation^20^, ENSO^21^ and sunspot numbers^8^, has been revealed.

However, few reports are available that addresses scale invariance and cross-correlation between streamflow and sediment load fluctuations, which are generally regarded as aggregate signals representing the overall hydrological alteration. Although the discharge is the main driver of the transport of sediment since the bulk annual variations of sediment load nearly matches that of the discharge, sediment storage is also an important control^22^. The complex governing processes of sediment dynamics are characterized by advection-diffusion mechanisms related to frequent settlement and resuspension. Sediments are thus stored during low discharge events and are delivered at high flow rates. The resistance and resilience of the sediment deposits are strongly steered by the flow field during flushing events^23^. In addition to flow capacity, the origin of sediment supply and homogeneity of sediment mixtures play an important role on the sediment transport. The type of hysteretical loop between flow and sediment depends on the availability of local in-channel stored sediment relative to the distal incoming sediment^6^, which is associated with the morphological evolution of the riverbed. These diverse physical mechanisms contributed to complex fluctuant features in time-varying sediment, which is probably different from streamflow fluctuation in the same channel reach. The objective of this study is therefore to characterize the multifractality of streamflow and sediment fluctuations and their multifractal response. Powerful tools including MF-DFA and MF-DXA allow for the exploration of multifractal structure and cross-correlation for concurrent streamflow and sediment.

## Field Site Location

**Figure 1:**
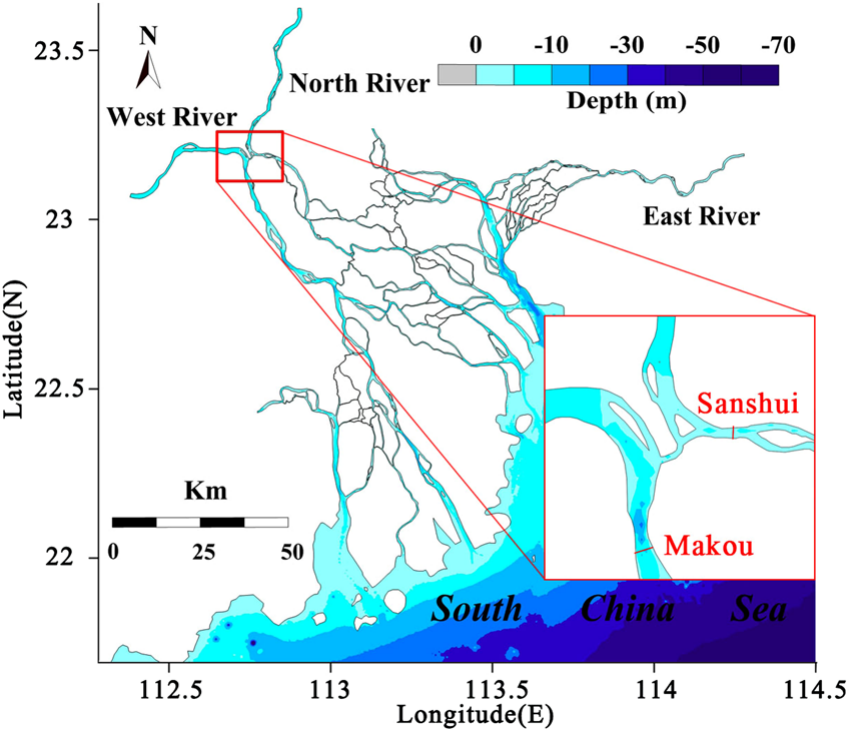
The map of Pearl River Estuary and the location of hydrological stations.

**Figure 2:**
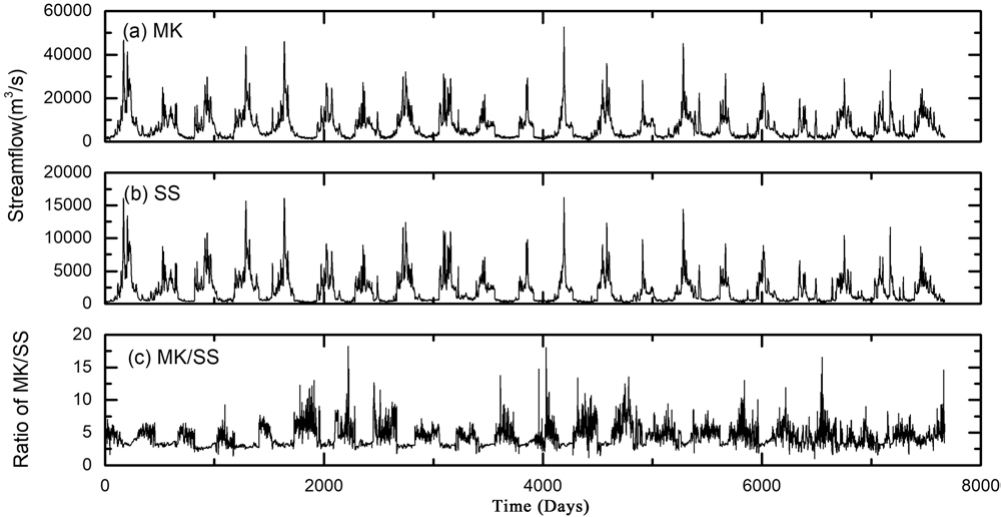
Temporal variations of daily streamflow at MK (**a**), SS (**b**) and the ratio MK/SS (**c**).

**Figure 3:**
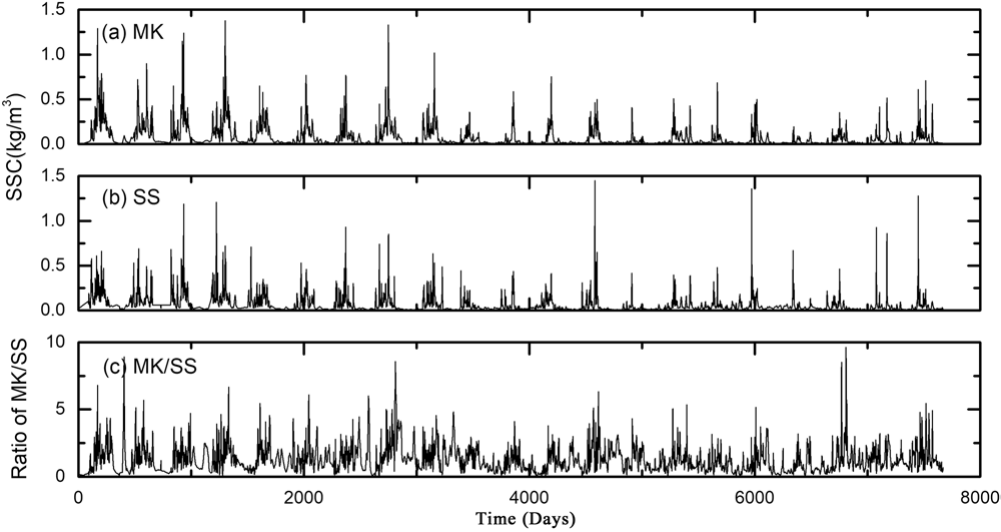
Temporal variations of daily SSC at MK (**a**), SS (**b**) and the ratio MK/SS (**c**).

The Pearl River is the second largest river in China, flowing through the highly economic development region. Its three main tributaries, namely West River, North River and East River, branch out into one of the most complex river networks in the world^24^ (Fig. 1). The tie channel of the West and North River is the apex of the Pearl River Delta and the first order bifurcation of the river network. The streamflow and sediment division at the bifurcation exerts a vital influence on the hydrodynamic and geomorphologic processes, fresh water offtake and channel navigability in the lower reach and the nearshore region^25^. The hydrological fluctuation associated with the multifractal behavior and long-range correlations thus needs to be explored in detail. In this study, two main gauging stations of the West and North River near the bifurcation, namely Makou (MK) and Sanshui (SS), are selected to characterize the flow and sediment fluctuation at the apex of the Pearl River Delta. Although the two hydrological stations are geographically close, their fluvial regimes primarily depend on the distinct characteristics of river basins, which provides an interesting case to explore different multifractal properties of hydrological signals in adjacent stations. Uninterrupted daily streamflow (Fig. 2) and suspended sediment concentration (SSC) (Fig. 3) were concurrently collected from 1994 to 2014 in Makou (MK), Sanshui (SS). The ratios between the values of the two gauging stations (MK/SS) were further calculated (Figs 2 and 3). Based on MF-DFA and MF-DXA, we decoded these data and characterized the multifractal properties and cross-correlation of the streamflow and sediment fluctuation.

## Analysis Techniques

Generally, natural time series are readily subjected to non-stationarities, including artificial noises and tendency, which may lead to unreliable or even spurious results in the process of data analysis^26^. Many intrinsic fluctuations, such as stock index in economy^27,28^, heart beat in biomedicine^16,29^, streamflow in hydrology^15,30^, plasma fluctuations in chemistry^31^ and cosmic radiations in physics^32^, exhibit substantially non-stationary behaviors. Multifractal Detrended Fluctuations Analysis (MF-DFA) has been widely applied to explore multifractal properties and scaling behaviors of nonstationary time series. Furthermore, Multifractal Detrended Cross-Correlation Analysis (MF-DXA) has been proposed to study the mutual influence with respect to two non-stationary time series^19^, which is similar to the MF-DFA. Suppose that *x*_*k*_ is a time series of length *N* for MF-DFA (*x*_*k*_ and *y*_*k*_ for MF-DXA). Both MF-DFA and MF-DXA procedures consist of five steps^33,34^:

- Step 1: Calculate the profile of time series as:

1 $$\begin{array}{l}{X}_{i}=\sum _{k=1}^{i}\,({x}_{k}-\langle x\rangle )\\ {Y}_{i}=\sum _{k=1}^{i}\,({y}_{k}-\langle y\rangle )\end{array}\,i=1,\ldots ,N$$

where $\langle x\rangle$ and $\langle y\rangle$ are the mean of *x*_*k*_ and *y*_*k*_, respectively.

- Step 2: Divide the profile *X*(*i*) and *Y*(*i*) into non-overlapping intervals *N*_*s*_ with equal timescale *s* (${N}_{s}=\,{\rm{int}}(N/s)$). Generally, a part of the data has the tendency to be left at the end of the profile when the length of time series cannot be an integer multiple of the timescale. Hence, for the purpose of not leaving out the aliquant part, it is necessary to repeat the same division procedure from the opposite end of the time series. This data processing method guarantees that 2 *Ns* non-overlapping segments are attained.
- Step 3: Determine the local trend for 2*Ns* segments with least squares fit method. The variance is defined as:

2 $${F}^{2}(s,v)=\frac{1}{s}\,\sum _{i=1}^{s}\,{\{X[(v-1)s+i]-{x}_{v}(i)\}}^{2}$$

3 $${F}^{2}(s,v)=\frac{1}{s}\,\sum _{i=1}^{s}\,\{X[(v-1)s+i]-{x}_{v}(i)\}\times \{Y[(v-1)s+i]-{y}_{v}(i)\}$$

For each segment *v* = 1, …, *Ns* (equation (2) for MF-DFA and equation (3) for MF-DXA), and

4 $${F}^{2}(s,v)=\frac{1}{s}\,\sum _{i=1}^{s}\,{\{X[N-(v-{N}_{S})s+i]-{x}_{v}(i)\}}^{2}$$

5 $${F}^{2}(s,v)=\frac{1}{s}\,\sum _{i=1}^{s}\,\{X[N-(v-{N}_{S})s+i]-{x}_{v}(i)\}\times \{Y[N-(v-{N}_{S})s+i]-{y}_{v}(i)\}$$

For each segment *v* = *Ns* + 1, …, 2*Ns* (equation (4) for MF-DFA and equation (5) for MF-DXA), where *x*_*v*_(*i*) and *y*_*v*_(*i*) are the fitting polynomial in segment *v*th. Any order polynomial can be applied to the fitting procedure.

- Step 4: Average the *q*th-order fluctuation function, *F*_*q*_(*s*) over all segments. *F*_*q*_(*s*) is expressed as:

6 $${F}_{q}(s)={\{\frac{1}{2{N}_{S}}{\sum _{v=1}^{2{N}_{s}}[{F}^{2}(s,v)]}^{q/2}\}}^{1/q}$$

where $q\ne 0$ and *s* ≥ *m* + 2. Generally, the variable *q* can take any real value apart from zero^15^. In terms of *q* = 2, the standard MF-DFA and MF-DXA are retrieved.

- Step 5: Calculate the slope of log-log plots of *F*_*q*_(*s*) versus *s*. The scaling exponent *h*(*q*) is described as:

7 $${F}_{q}(s) \sim {s}^{h(q)}$$

The slope of log-log plots of *F*_*q*_(*s*) versus *s*, *h*(*q*), is the generally Hurst exponent. The *h*(*q* = 2) is known as the Hurst exponent for stationary data. Furthermore, the dependence of *h*(*q*) on *q* indicates the multifractal behavior of time series, while the monofractal data behave as independence of *h*(*q*) on *q*. In terms of positive *q*, the scaling behavior of the segments with huge fluctuations is characterized by the general Hurst exponent. However, negative *h*(*q*) characterizes the scaling behavior of the segments with mild fluctuations^30^.

Additionally, the mass exponent function $\tau (q)$ can be calculated by the generalized Hurst exponent *h*(*q*):

8 $$\tau (q)=qh(q)-1$$

Furthermore, the singularity spectrum *D*(*α*), can be derived by conducting first-order Legendre transformation:

9 $$D(q)=q\alpha -\tau (q)$$

where $\alpha =\tau ^{\prime} (q)$. The relationship between the singularity spectrum *D*(*α*) and the singularity exponent *α* can be expressed by integrating Equation (7):

10 $$D(q)=q[\alpha -h(q)]+1$$

The width of the singularity spectrum (${\rm{\Delta }}$*α* = *α*_*max*_ − *α*_*min*_) implies degree of multifractality of the time series, which is related to the dependence of *h*(*q*) on *q*. The singularity spectrum collapses a single point so the width of the singularity spectrum will be zero, indicating a monofractal series. Accordingly, *h*(*q*) is independent of *q*. The wider the singularity spectrum, the more multifractal is the spectrum.

## Results

### Multifractal Detrended Fluctuation Analysis

**Figure 4:**
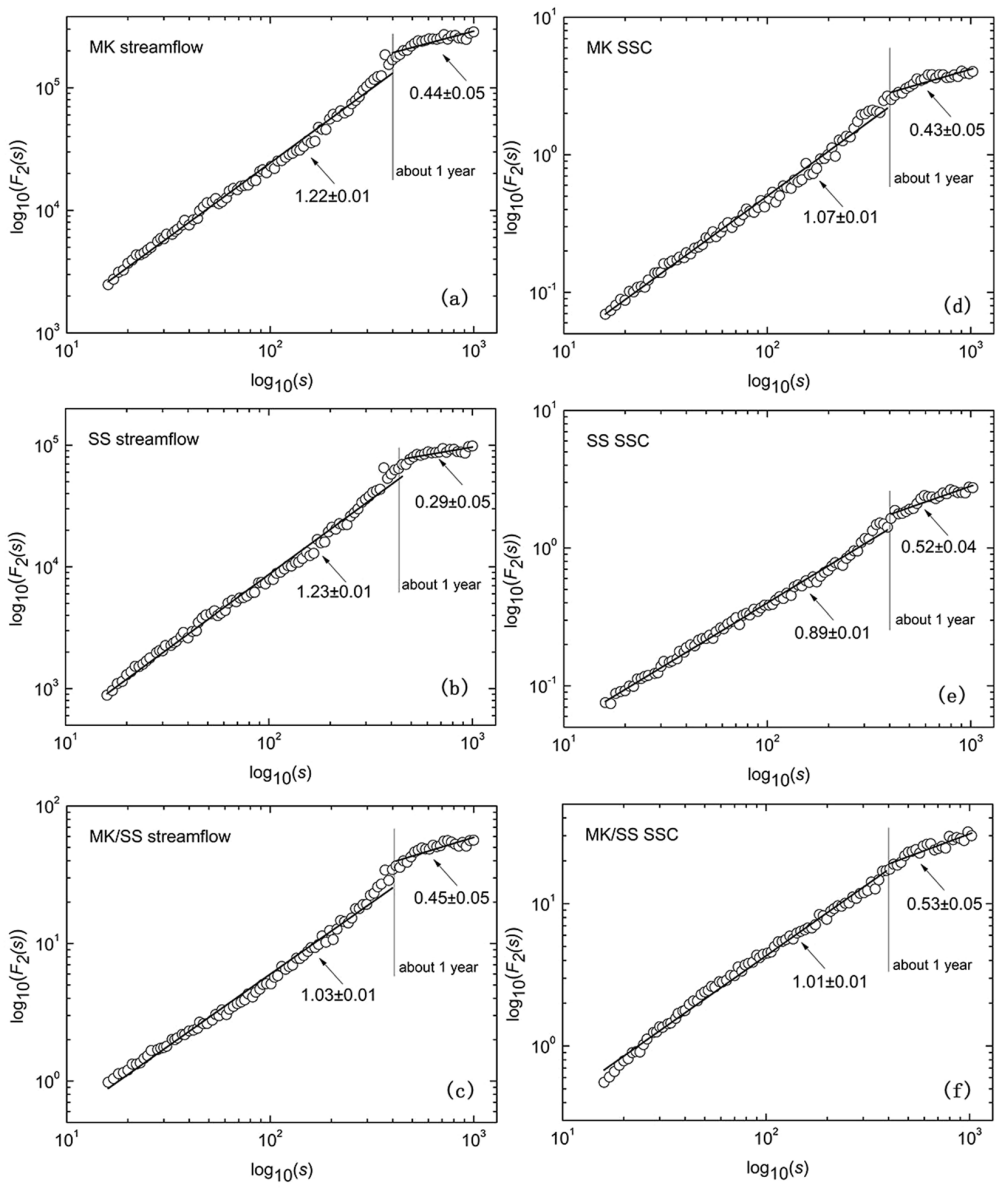
Scaling properties of log-log plots of *F*_*2*_(*s*) versus *s* of daily streamflow (left column) and SSC (right column) of MK (top row), SS (middle row) and MK/SS (bottom row). The slope values and associated errors of the fitting lines have been displayed.

The scaling behavior of the streamflow and SSC at MK and SS is illustrated in Fig. 4. It is obvious that crossover points with about one-year can be found in all log-log plots of *F*_*2*_(*s*) versus *s* of time series. This may be an indication of the annual periodicity and the competition between noise and sinusoidal trend^35^. The curves of the log-log plot of *F*_*2*_(*s*) versus *s* are divided into two scaling regions by crossover points, both of which are fitted by numerical techniques (Fig. 4). Below the crossover, the slope values of streamflow log-log curves at MK and SS are nearly equal on small timescale (1.22 ± 0.01 for MK and 1.23 ± 0.01 for SS), which implies similar periodicity properties. The intercept values of the fitted lines are obviously different, indicating distinct changing magnitude of streamflow between MK and SS. For large timescale of more than one year, the slope value of streamflow fitted line at MK is 0.44 ± 0.05, which is relatively larger than the slope value at SS (0.29 ± 0.05). Furthermore, the slope value of SSC fitted line at MK (1.07 ± 0.01) is larger than that at SS (0.89 ± 0.01) at the shorter time scale. In contrast, the slope value at MK (0.43 ± 0.05) is smaller than SS (0.52 ± 0.04) at large timescale, implying that the scaling behavior of SSC at SS is relatively more consistent than MK station. Interestingly, scaling properties between the MK/SS ratios of the streamflow and SSC are nearly uniform. The slope value of streamflow ratio at MK (1.03 ± 0.01) is close to the slope value at SS (1.01 ± 0.01) for the time scale less than one year. Meanwhile, larger difference is found for the time scale of more than one year (0.45 ± 0.05 at MK and 0.53 ± 0.05 at SS).

**Figure 5:**
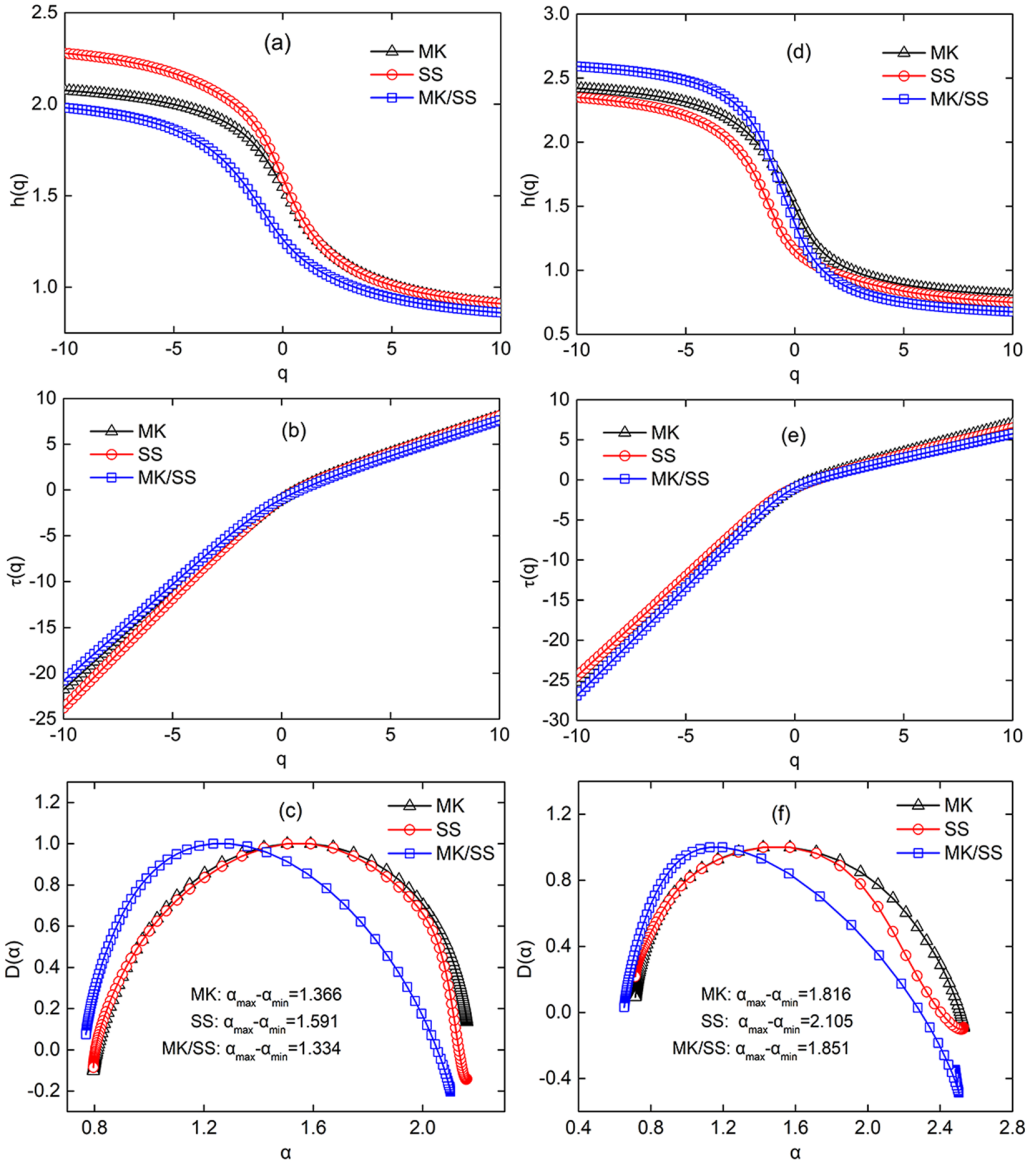
The multifractal spectrums of streamflow (left column) and SSC (right column) at MK, SS and MK/SS. The relationships of (1) the generalized Hurst exponent *h*(*q*) and *q* (top row); (2) the mass exponent function *τ*(*q*) and *q* (middle row); and (3) the singularity spectrum *D*(*α*) and singularity exponent *α* (bottom row).

The multifractal spectrums of daily streamflow and SSC are displayed in Fig. 5. Figure 5(a,d) clearly exhibit that the generalized Hurst exponents, *h*(*q*), vary with *q* of the streamflow and SSC series, suggesting the multifractal properties as indicated by predominant *q*-dependence of *h*(*q*). Furthermore, different relationships between the mass exponent function *τ*(*q*) and *q* for −10 < *q* < 0 and 0 < *q* < 10 can be found in Fig. 5(b,e). The slope values of *τ*(*q*) versus *q* are fitted and showed in Table 1. The degree of the nonlinearity of the mass exponent function *τ*(*q*) virtually implies the degree of multifractal behaviors^36^. The difference between the slope values of *q* < 0 and *q* > 0 for SSC is substantially larger than streamflow series, indicating that the SSC possesses higher multifractality. The *τ*(*q*) of the ratio MK/SS in SSC (2.02) shows the largest difference between the slope values of *q* < 0 and *q* > 0, while the *τ*(*q*) of the ratio MK/SS in streamflow is the smallest (1.20), indicating significantly distinct multifractality between the allocation of streamflow and SSC at MK and SS.

**Table 1:** Slope values of mass exponent function *τ*(*q*) for daily streamflow and SSC of MK, SS and the ratio MK/SS.

	Streamflow			SSC		
	MK	SS	MK/SS	MK	SS	MK/SS
−10 < q < 0	2.10	2.32	2.03	2.48	2.43	2.66
0 < q < 10	0.86	0.86	0.83	0.76	0.72	0.64

The asymmetric singularity spectrum *D*(*α*) and singularity exponent *α* of the time series are displayed in Fig. 5(c,f). The singularity spectrum with a long right tail and left tail reflects that the multifractal structure of time series is sensitive to the local fluctuations with large magnitudes and to those with small magnitudes, respectively^37^. The singularity spectrum of streamflow at MK is characterized by right truncation, whereas the singularity spectrum of streamflow at SS is by left truncation. Furthermore, pronounced left truncations exist for the singularity spectrum of the ratios MK/SS in streamflow and SSC, which means that the ratios MK/SS in streamflow and SSC are sensitive to the small magnitude of local fluctuations. The multifractal spectrum widths (*α*_*max*_-*α*_*min*_) of SSC are significantly larger than streamflow series, implying that the temporal fluctuations of SSC present high degree of multifractality. Moreover, the singularity spectrums of streamflow and SSC at SS show a wider range than MK and MK/SS.

### Multifractal Detrended Cross-Correlation Analysis

**Figure 6:**
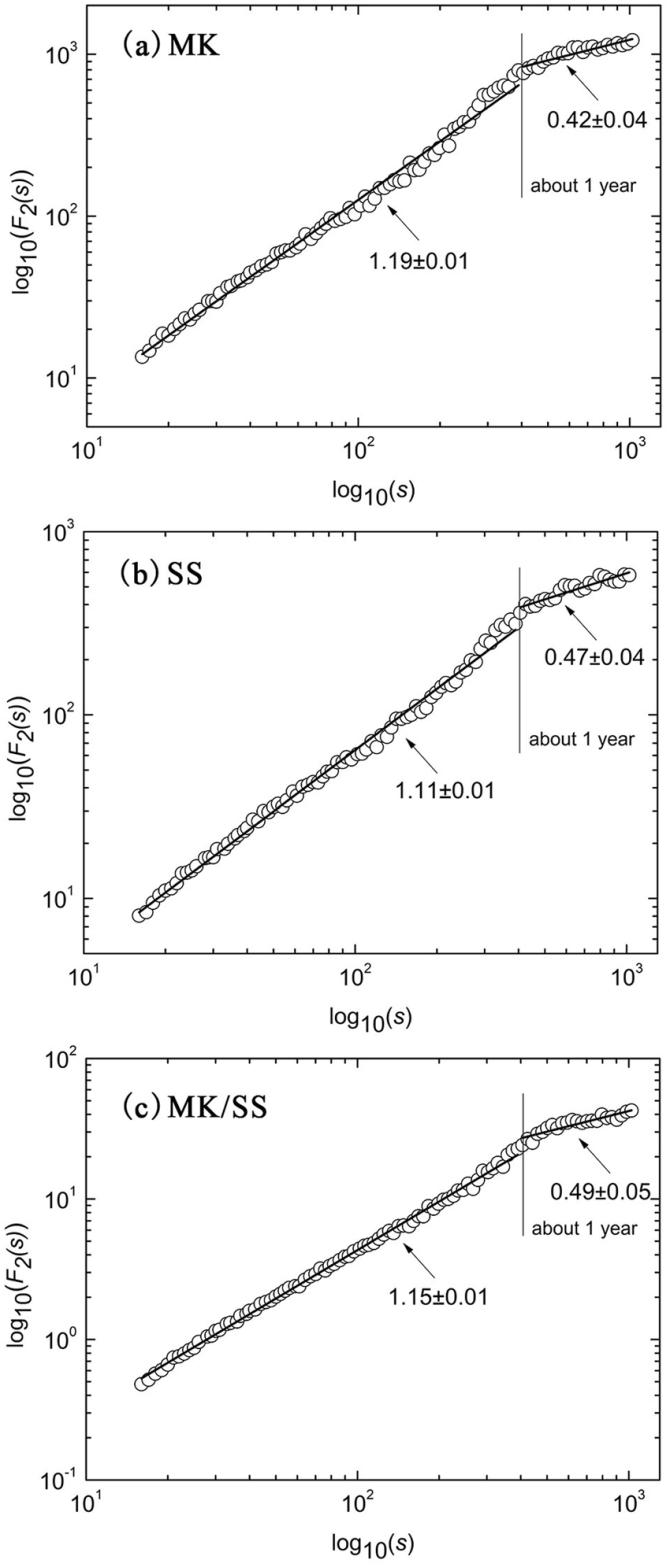
The log-log plots of fluctuation function given by the MF-DXA of daily streamflow and SSC at MK (**a**), SS (**b**), and MK/SS (**c**).

The MF-DXA procedure is employed to detect the multifractal properties and the cross-correlation of streamflow and SSC fluctuations (Fig. 6). The time series are synchronized to attain reliable results. Log-log plots of *F*_*2*_(*s*) versus *s* introduced by MF-DXA for streamflow and SSC series are characterized by two scaling regions, which display the multi-scale response of streamflow and SSC. About one-year crossover divides the scaling regimes into small and large periods. Linear fittings are conducted on different scaling regions to acquire scale exponents.

On small timescale, the scaling exponents for MK, SS and MK/SS correspond to 1.19 ± 0.01, 1.11 ± 0.01 and 1.15 ± 0.01, respectively. All of them are larger than 1, implying positive long-range correlation between streamflow and SSC. SSC has the tendency to increase (decrease) synchronously with the increasing (decreasing) load of streamflow. For the large scale, the scaling exponents of MK, SS and MK/SS are 0.42 ± 0.04, 0.47 ± 0.04 and 0.49 ± 0.05, respectively. The scaling exponents are close to 0.5, indicating a random behavior with no obvious correlations between streamflow and SSC fluctuations for the time scale larger than one-year. The dominant long-range correlation shifts to random behavior or slightly anti-persistent long-range correlation as the time scale increases.

## Discussion

### The causes of multifractality

There are, in general, two main reasons that may result in the multifractal behaviors of hydrological data^15,38^: (1) the broadness of probability density function (PDF) of the time series and, (2) the long-range correlations of the time series, which are known as the Noah phenomenon and the Joseph phenomenon, respectively^39^. In order to distinguish the type of multifractality of the hydrological data, the shuffled and surrogate methods could be employed. The surrogate data could maintain different correlations in small and large-scale fluctuations while the broadness of PDF shifts to Gaussian distribution. In contrast, the shuffled data would be able to remove the multifractality caused by the long-range correlations without affecting the multifractal behavior caused by the broadness of PDF. If the multifractality of the hydrological data is solely caused by the broadness of PDF, then the generalized Hurst exponent *h*(*q*) derived from the surrogate data should have the potential to be *q*-independent. Also, the generalized Hurst exponent *h*(*q*) obtained by the shuffled procedure would be equal to 0.5 when the long-range correlations exert an overwhelming impact on the multifractality of the hydrological data. Weaker multifractality would be found in both surrogate and shuffled data if two types of causes are responsible for the multifractal behavior. The surrogate and shuffled data are thus applied to explore the causes of multifractality.

**Figure 7:**
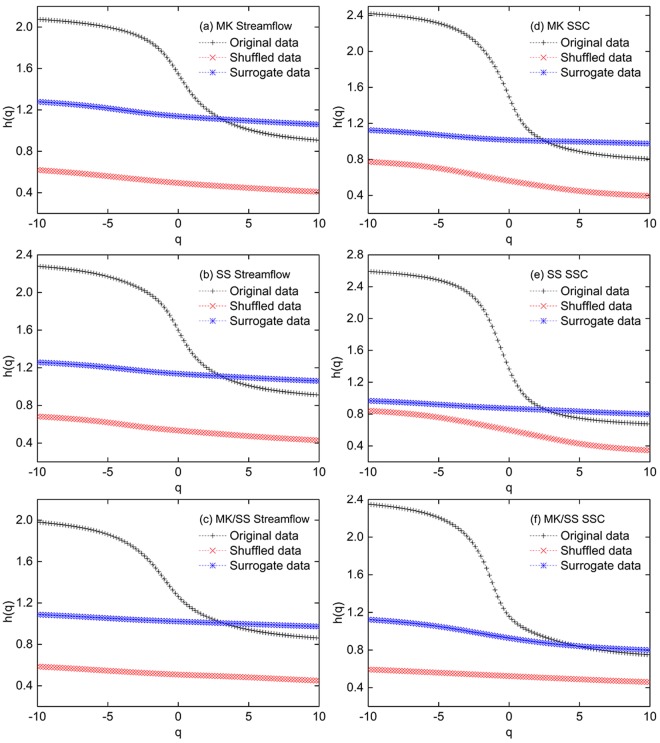
The generalized Hurst exponent *h*(*q*) verse *q* for the streamflow (left column) and SSC (right column) at MK, SS and MK/SS for original, shuffled and surrogate data.

It is obvious that the multifractality of the surrogate and shuffled data of streamflow and SSC for MK, SS and MK/SS is substantially weaker in comparison to the original data (Fig. 7). The multifractality of hydrological data thus is caused by both the long-range correlations and the broad probability density. The generalized Hurst exponents *h*(*2*) of the shuffled data for all time series roughly equal to 0.5, but the *h*(*q*) is dependent on the *q* and exhibits a monotonically decreasing trend, which indicates the broad probability density is responsible for the multifractal behavior. Similarly, the curves of surrogate data, which is higher than the shuffled data, also show no substantial independence of the *h*(*q*) values on *q*, indicating the multifractality is partly caused by the long-range correlations of the hydrological data. To further identify the contribution of the correlation and the broadness of PDF, the multifractal spectrum widths (${\rm{\Delta }}$*α* = *α*_*max*_ − *α*_*min*_) of the surrogate and shuffled data are applied to evaluate degree of multifractality. The ${\rm{\Delta }}$*α* values of shuffled and surrogate date are significantly less than the original ${\rm{\Delta }}$*α*, which corroborates that both the correlation and the broad probability density lead to the multifractality (Table 2). The ${\rm{\Delta }}$*α* of surrogate data for streamflow at MK, SS and the ratio MK/SS are 0.566, 0.478 and 0.279, respectively, which are larger than the corresponding shuffled data, implying that the impact of the correlation on the multifractal behavior is relatively stronger than the broadness of PDF for streamflow data. Conversely, for SSC at MK and SS, since the shuffled ${\rm{\Delta }}$*α* values are larger than the surrogate ${\rm{\Delta }}$*α* values, it can be concluded that the broadness of PDF plays a dominant role in the multifractality. However, opposite characteristics are found on the ratio MK/SS in SSC. The influence of the correlation is relatively more pronounced than the broadness of PDF as the surrogate ${\rm{\Delta }}$*α* (0.367) is larger than the shuffled ${\rm{\Delta }}$*α* (0.238).

**Table 2:** The multifractal spectrum widths (${\rm{\Delta }}$*α* = *α*_*max*_ − *α*_*min*_) of the streamflow and SSC at MK, SS and the ratios MK/SS.

Data	Streamflow			SSC		
	MK	SS	MK/SS	MK	SS	MK/SS
Original	1.366	1.591	1.334	1.816	2.105	1.851
Shuffled	0.361	0.405	0.257	0.532	0.695	0.238
Surrogate	0.566	0.478	0.279	0.269	0.314	0.367

### The implication of multifractal properties

The multifractal properties of the hydrological data are subjected to the climate fluctuation^3,8^, anthropic interferences^40–42^ and the characteristic of drainage basin, such as topography^43^, land cover^44^, drainage area^45^. Generally, hydrological processes are highly vulnerable to the changing pattern of precipitation. Although the seasonal and annual variations of streamflow is mainly influenced by the precipitation changes, the long-term persistence of the streamflow is barely related to the persistence in precipitation^3^. Based on the long daily records from 99 meteorological stations and 42 hydrological stations in the world, Kantelhardt demonstrated that the fluctuation exponents of streamflow varied in a broad range, indicating significant autocorrelation, while precipitation records displayed a fast decay of the autocorrelation function.

Intensive human activities have significantly changed the hydrological regime in the last several decades, especially reservoirs constructions. Reservoirs are constructed for navigation, irrigation, energy production and providing safety against flood and drought events. Coarser sediments have the potential to settle down with the decreasing flow velocity and transport capacity when incoming discharges and sediments are trapped, while the fine sediment storage is removed by reservoir release and flushing. The annual cycle of storage and release is closely intertwined with the multifractal response between streamflow and sediment fluctuations, indicating the long-range correlation on less than one-year timescale. However, the prevalence of reservoirs sedimentation can give rise to continuous decrease of sediment load and clogging the outlet structure, which generally exerts detrimental impacts on the coherent continuum of natural sediment cycle^46^. Additionally, the upstream reservoirs entrapment also modulates the distal sediment supply and sediment size related to different hysteresis types of streamflow and sediment^6^. These patterns may account for the out-of-phase response between streamflow and sediment fluctuations.

For the West River, the reservoir’s storage capacity was about 38.4 × 10^9^ m^3^, which was much higher than the storage capacity at the North River (6.6 × 10^9^ m^3^)^47^. Thus, to a certain degree, tiny streamflow and sediment fluctuations are virtually eliminated due to reservoir storage^40^, which sheds some light on higher multifractal degree of streamflow and sediment at SS. Furthermore, although reservoir storage associated with river regulation has the potential to flatten the intra-annual fluctuation of hydrological signals, the seasonal difference of sediment is larger than streamflow. Similar phenomenon can be found in the East River, the most regulated tributary of the Pearl River^48^. Zhang corroborated that the intra-annual distribution of sediment was much more uneven than streamflow. These results indicate higher degree of multifractality in sediment fluctuation, which is accordant well with the finding in the previous section (Fig. 5).

Apart from the reservoir storage, the characteristics of drainage basin can be also incorporated to interpret the difference of multifractal degree. On one hand, the storage processes in the soil, which is related to the buffering effect, have been corroborated to control the long-term persistent fluctuations of hydrological data. In this study, most of the streamflow and sediment at MK and SS come from the upper reaches of the West and North River, which is characterized by distinct land covers and soil types. The West River, the primary tributary of the Pearl River, flows through the largest karst rocky desertification region in the world^47^. The weathered limestone plays a dominant role at the West River basin while the geomorphological types at the North River basin consist of several rocks, including shales, granite and clastic rocks^49^. The different origins of sediment related to nonuniform sediment mixtures and grain sizes at the West and North River basins may give rise to distinct cross-correlation and hysteresis types between flow and sediment^6^. Furthermore, vulnerable land types aggravate soil erosion at the West River basin, leading to the failure of water and sediment maintenance and the mitigation of the buffering effect, while better water and soil conservation are found at the North River basin. Therefore, the distinct multifractality and persistence of streamflow and sediment at MK and SS can be partially interpreted as the upstream land cover. On the other hand, the different multifractal spectrum widths at MK and SS also depend on drainage areas of the West and North River. Based on the analysis of 18 typical rivers in Germany and 23 international rivers, Koscielny discovered that the multifractal width, as the “fingerprints” of the hydrological data, decreased slightly with the increase of drainage area^45^. According to the daily records from five river basins, Özger further corroborated that larger basin areas had the potential to present higher persistence structure and smaller multifractality^26^. In this study, the drainage area of the West River is 30.5 × 10^4^ km^2^, which is considerably larger than the North River (5.2 × 10^4^ km^2^). This may partially reveal that degree of multifractality of both streamflow and sediment at SS is discernibly stronger than that at MK.

## Conclusions

Based on long daily streamflow and sediment data at the apex of the Pearl River Delta, the multifractality and multi-scale cross-correlation between the streamflow and sediment were explored by the MF-DFA and MF-DXA. Due to the annual periodicity and the competition between noise and sinusoidal trend, about one-year crossover points are prevalent in all log-log plots of *F*_*2*_(*s*) versus *s* of time series. Although the scaling properties of streamflow between MK and SS are almost identical on less than one-year scale, evident divergences of the scaling exponents occur in the SSC fluctuation. Interestingly, despite distinct scaling exponents between streamflow and SSC fluctuation at MK and SS, the ratios MK/SS in streamflow and SSC have the tendency to present similar periodicity properties and fluctuation behavior at full scales. Moreover, the singularity spectrums display pronounced left truncations, implying the ratios MK/SS in streamflow and SSC are sensitive to the small magnitude of local fluctuations.

Similar crossover points were found on the multi-scale cross-correlation analysis between streamflow and SSC. The scaling exponent is larger than 1 at small timescale while it is close to 0.5 for the large timescale, which indicates the dominant long-range persistent shifts to random behavior or slightly anti-persistent long-range correlations as the time scale increases. Additionally, the multifractal spectrum widths of surrogate and shuffled data were compared with the original data to evaluate the contribution of the broadness of PDF and the long-range correlations. The multifractal spectrum widths of both shuffled and surrogate data are significantly undermined, implying that the integrating impacts of broad probability density and long-range correlations should be responsible for the multifractality. The long-range correlations play a dominant role on the multifractal behavior for streamflow at MK and SS with larger surrogate ${\rm{\Delta }}$*α*. In contrast, the multifractal behavior of SSC fluctuation depends heavier on the broadness of PDF. Although two hydrological stations are adjacent in terms of geographical location, the temporal fluctuations of streamflow and SSC at SS present higher multifractal degree and complexities than at MK, which can be partly contributed to the much larger drainage area of the West River. Furthermore, distinct land covers and the magnitude of anthropic interference, especially reservoirs constructions, should be incorporated to explain the difference in multifractal structure at West and North River basin.
